# Economic Growth, People's Livelihood Preferences of Local Governments and Residents' Health

**DOI:** 10.3389/fpubh.2022.844015

**Published:** 2022-04-08

**Authors:** Shu-tian Cen, Wei-hai Yan

**Affiliations:** ^1^Shanghai Jinshan District Party School of CPC, Shanghai, China; ^2^Guangxi University of Chinese Medicine, Nanning, China

**Keywords:** health policy, economic growth, residents' health, local government behavior, people's livelihood preferences

## Abstract

This article is aimed to examine the effect of economic growth on the health of residents from the perspective of local government behavior. First of all, it is theoretically proved that in an economy with economic decentralization and political centralization, because the local government implements the central planner's “people-oriented” and “people-centered” requirements, the local government pays attention to both its own consumption and the health of residents. For this reason, he prefers public health investment, and the conclusion is that there is a stable and balanced relationship between residents' health and economic growth. When the local government's relative importance to people's livelihood is greater than its own consumption, the economic growth will have a significant and positive impact on residents' health. And the internal mechanism of economic growth affecting residents' health is combed mathematically. Secondly, using panel data from China's 31 inland provinces from 2003 to 2019, empirical tests show that the hypothesis that China's economic growth promotes residents' health is established. The work of this article means that the improvement of residents' health depends not only on the total amount of available resources brought about by economic growth, but also on the government's livelihood preferences. The public health investment behavior of local governments in China is an important clue to explains that China's economic growth can promote residents' health.

## Introduction

Since 1978, the health level of Chinese residents has continued to improve, and the average life expectancy has continued to increase. It was 67.77 years old in 1981, 71.4 years old in 2000, 76.34 years old in 2015, and 77.3 years old in 2019^1^. At the same time, China's economy achieved rapid and sustained growth during the period from 1978 to 2012, with an average annual GDP growth rate of over 9.5%, which was known as “China's growth miracle” by the world. Although China's economy has entered a new normal state and is in a stage of high-quality development after 2012, its performance is still outstanding in the world, especially in the context of the COVID-19. Then, whether there is a relationship between China's economic growth and residents' health, what is the effect, what is the internal mechanism, and what role the government plays in residents' health have become important research propositions. In fact, the government plays an important role in two aspects of residents' health behavior: one is to govern environmental pollution caused by economic growth, to improve environmental quality, and to reduce the damage caused by environmental pollution to residents' health; the other is to invest in public health facilities, improve the accessibility and quality of medical services, and enhance the protection of residents' health. We call these investments in public health investment. If the government spends more resources on these two aspects, the greater the positive impact of economic growth on residents' health should be.

**Figure 1:**
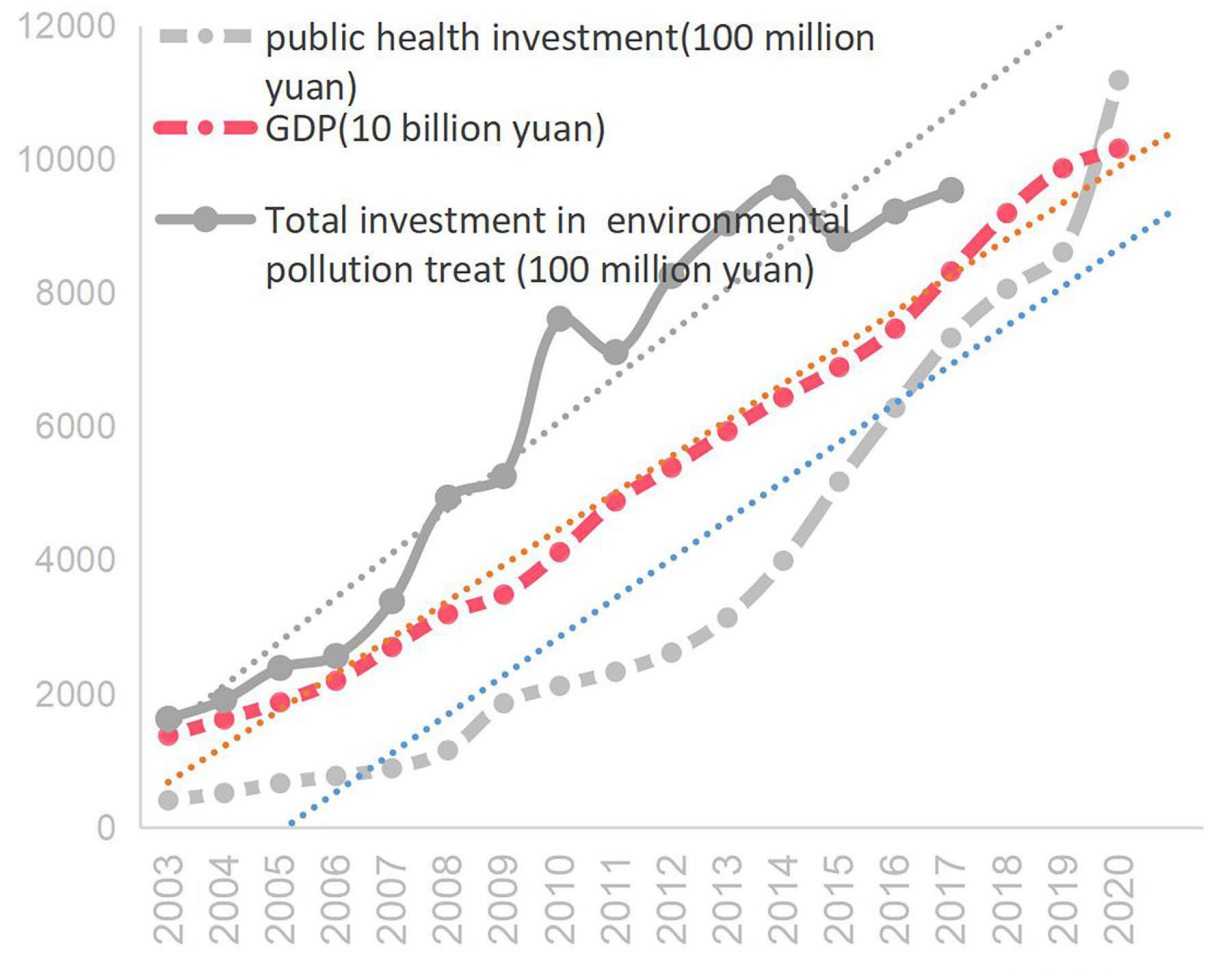
China's public health investment, treat environmental pollution investment and GDP. *Source:* National data from the National Bureau of Statistics of China^3^.

Since the beginning of the new century, local governments at all levels in China have thoroughly implemented the “people-oriented” scientific development concept and the “people-centered” development philosophy, and attached great importance to public health investment^2^. It can be seen from Figure 1 that since 2003, China's investment in environmental pollution control, investment in public health facilities, and GDP have maintained roughly the same growth rate. The trend line of environmental pollution treat investment and the GDP trend line show a “micro-scissors difference,” indicating that the growth rate of environmental pollution control investment has surpassed GDP growth, and the investment in public health facilities is basically parallel to the GDP trend line, indicating that the growth rate of investment in public health facilities is basically consistent with GDP growth. Existing research shows that the improvement of residents' health level depends not only on available resources, but also on resource allocation. Even if the economic level is low, by allocating more resources to health services, the gradual improvement in health can also be ensured. Cuba is a typical case. In the absence of economic development, policy support has ensured people's health demands and improved residents' health (2). This is essentially due to the government's livelihood preferences.

The organization of this paper are as follows. Section Literature Review reviews the existing literature. Section Dynamic Equilibrium Model constructs a theoretical model of local government's livelihood preference behavior, and derives a stable and balanced relationship between the residents' health level and regional economic growth. Section Empirical Analysis is empirical test based on the theory of the third section and uses the 31 province-level panel data of China. The fifth section is the conclusion.

## Literature Review

Research on the relationship between economic growth and residents' health has always been a hot topic. The existing literature on health economics focuses on the contribution of healthy human capital to economic growth. Since the NBER Human Capital Conference and the paper of Gary Becher's Human Capital Investment is published, people have gradually realized that health, as an important human capital, has a particularly significance for economic growth (3, 4), that is, the impact of residents' health on economic growth is generally positive. The literature of this type of research is abundant. But another aspect of the coin, the impact of economic growth on the health of residents, is much less concerned. One view is that as long as economic growth goes up, other aspects including health will naturally go up (5). This view holds that economic growth can improve the living standards of residents and improve the quality of life, which is of course conducive to promoting health and longevity. But this is not the case. There has been an endless debate on the topic of whether economic growth has promoted the health of residents, and continues to this day. The impact of economic growth on the health of residents will vary depending on the region, the country, the stage of economic development, the type of disease, the gender, and the age (6–8), the differences are extremely large, and even the conclusions are opposite, with a high degree of inconsistency.

First, channels for economic growth to improve residents' health are existing, including economic growth can improve residents' food, clothing, housing and transportation, improve people's immunity, and thereby improve residents' health; economic growth promotes advancement in health technology, and effective control of the incidence and mortality of infectious diseases; economic growth significantly reduces gender, ethnic or religious discrimination, Improve the level of education, popularize health knowledge, continuously improve the health literacy of residents, and promote the overall improvement of health; economic growth accelerates the process of urbanization, increase investment in public health, improve the accessibility and quality of medical services, etc. (8).

Second, channels which economic growth negatively affects the health of residents are also existing, including the direct mechanism, such as economic growth leading to pollution, over-urbanization, and economic resources occupying people's livelihood resources, especially extensive economic growth, which often leads to problems such as high pollution, excessive use of natural resources, and lack of sanitary conditions; economic growth leads to individual jobs time increased, and extended working hours have increased the opportunity cost of residents to engage in healthy and leisure activities, reduced social interaction, and squeezed sports time; economic growth has also increased official and travel, which has also increased the incidence of traffic accidents; economic growth also increases personal work intensity and creates additional work pressure, and it can also lead to poor health behaviors such as lack of sleep, poor mood, alcoholism, smoking, and reduced exercise, leading to a decline in health, and work pressure of parents will also form an intergenerational effect, which negatively affects the health of the next generation; people's behavior and health awareness cannot quickly adapt to changes in the external environment brought about by economic growth, leading to chronic diseases surge such as obesity, diabetes, hypertension, cardiovascular disease by high-calorie consumption (9–15). Also including the indirect mechanism, such as changes in the social environment, economic growth affects the surrounding environment and then affects the health of residents; the decline in economic growth has changed the whole the social environment, in a social environment where the number of unemployed people continues to increase, the decline in health caused by unemployment has increased (7); the degree of completeness of the social security system is important, facing the same degree of economic downturn, the health of residents which countries with a more complete social security system is well protected and prevented from falling, for example, during the Great Depression in the United States, the health of residents in cities with a relatively sound social security system was not negatively affected by the economic downturn (16).

Third, there are some indications that China's economic growth affects residents' health, such as China's economic growth improves the performance of residents' health. There is a positive correlation between economic growth and residents' health (17). The long-term effect of GDP on residents' health is positive, and health investment has effectively promoted the improvement of residents' health, and the correlation between the two has been continuously strengthened (18). In the long run, it is feasible to rely on promoting economic growth or increasing health input to improve the health of the people. The improvement of health will in turn promote the increase of health investment and promote economic growth, which will form a virtuous circle of mutual promotion; In the short term, the impact of economic growth on health is relatively obvious (19). There is a positive relationship between economic growth, health investment, and people's health. Economic growth brings more investment in health care, and the improvement of health service quality provides people with better medical environment and conditions, and the safe and healthy of people themselves can bring more economic development momentum, and the three are complementary and indispensable (20).

On the other hand, economic growth may lead to health deterioration in China. The original health problems have not been completely resolved, and new health problems such as cardiovascular and cerebrovascular diseases, cancer, chronic respiratory diseases, diabetes and other chronic non-communicable diseases have emerged with high incidence, and certain health indicators have not even increased but decreased. In China, undernutrition and overnutrition coexist at the same time, and the proportion of undernutrition remains high, but the problem of overnutrition has gradually become prominent, such as the increasing number of obese people. Major public health incidents continue to invade people's health. Since 1999, the fatality rate of Class A and B statutory infectious diseases has shown an overall upward trend year by year, with SARS in 2003 and COVID-19 in 2020. The possible reasons for these problems include the direct mechanism and the indirect mechanism. The direct mechanism included the widening income gap, environmental pollution, population mobility caused by urbanization, changes in lifestyle and other factors caused by the process of economic growth, which have adverse effects on health. For example, with the income gap widens, the marginal effect of income increases in improving the health of residents is diminishing, which will have a negative impact on residents' health (16, 21). Economic growth creates the environment Changes, especially industrial pollution, have a significant negative impact on residents' health, making the improvement of health lagging behind the level of economic growth. Economic growth has promoted the development of urbanization, resulting in a large number of population movements, and giving birth to serious impacts in family planning and the spread of diseases, especially the prevalence of intestinal infectious diseases, which has brought negative impacts on the health of residents. Along with economic growth, some unhealthy lifestyles and personal behaviors have also increased, and the dangers of diseases caused by smoking and drinking are increasing, and the incidence of bronchial lung cancer and alcoholic liver disease in our country is increasing (22). For the indirect mechanism, existing research focuses on analysis at the policy level. Insufficient financial investment and the disintegration of rural medical care are important reasons that caused the health level to lag behind economic growth in the early stage of reform and opening up (23). The imperfection of the medical care system reduces the health promotion effect of health expenditures to a certain extent (24).

In summary, through the combing of the status quo of research on the relationship between economic growth and residents' health, the current research has been relatively mature, presenting multiple perspectives and multiple dimensions. However, the existing research on the effects of economic growth on the health of residents is limited to empirical analysis in China^4^, lacking theoretical support and stable mathematical relationship analysis, and it is necessary to further clarify the internal mathematical mechanism of economic growth on the health of residents. On the other hand, the existing research is limited to the material relationship analysis, which ignores the positive role of government behavior in the analysis of the relationship between economic growth and residents' health. In fact, the most important point of why China's economic growth can promote the health of residents is that the Chinese government, including the central government and local governments, spend a large part of the limited fiscal revenue on public health investment, including public health facilities and environmental pollution Disposing. This provides space for follow-up research on this topic. This study attempts to make some efforts in these areas, and examines the relationship between China's economic growth and the health of residents in the new century from the perspective of local government behavior. First of all, it is theoretically proved that there is a stable equilibrium relationship between economic growth and residents' Health in an economy with economic decentralization and political centralization, as local governments implement the central planner's “people-oriented” and “people-centered” requirements.

Second, empirical tests show that the hypothesis that China's economic growth promotes the improvement of residents' health is established. This study means that the improvement of residents' health depends on both the total amount of available resources brought about by economic growth and the allocation of available resources by the government. The public health investment behavior of local governments in China is an important clue to explains that China's economic growth can promote residents' health.

## Dynamic Equilibrium Model

This section focuses on the impact of economic growth on residents' health based on the livelihood preferences of local officials.

### Model Environment and Basic Assumptions

The model draws on the framework of Acemoglu (25) and Cen (26) on public capital and economic growth, and Grossman (3, 4) on healthy human capital. It examines the impact of economic growth on residents' health based on the livelihood preferences of local officials in a politically centralized and economically decentralized economy. Specifically, consider an economy that includes healthy human capital and public health capital. It consists of N regions, each region has a local government, and there is only one central planner. Local government is the main body^5^ of public health investment in the region, and public health investment mainly depends on local financial investment^6^. Local governments consider consumer responses to policy parameters.

As representatives of local governments, local officials have two incentive preferences: local officials not only pursue the consumption maximization of local governments which is usually related to the economic incentives of local officials,^7^ but also implement the central planner's requirements of “people-oriented” and “people-centered,” and pursue basic maximize investment in regional public health^8^. The public health investment behavior of local officials mainly depends on the incentive motives of local officials, and change into its strength is usually related to the people's livelihood preferences of local officials. For this reason, when other conditions remain unchanged, the people's livelihood requirements of the central planner will have an important impact on the public health investment behavior of local officials.

Production function. There is a representative firm in area *i*, and its production function is:

(1) $$\begin{array}{r} Y_{t}^{i}\;=\;B_{t}^{i}{\left(K_{t}^{i}\right)}^{\alpha }{\left(M_{t}^{i}\right)}^{\omega }{\left(A_{t}^{i}\right)}^{\varphi } \end{array}$$

Where formula (1) is the C-D production function, $Y_{t}^{i}$ is output, $K_{t}^{i}$ represents corporate capital, $M_{t}^{i}$ represents healthy human capital^9^, and $A_{t}^{i}$ represents public health input capital (including environmental facility input); *t* is period, *i* is region, *i* = 1,2, …, N; α > 0, ω > 0 and φ > 0 represent output elasticity related to corporate capital, healthy human capital, and public health capital respectively, and α + ω + φ < 1^10^.

The decision of healthy human capital. It can be seen from formula (1) that healthy human capital (ignoring education human capital) is essential for the final product of society (3, 4). It is assumed that healthy human capital is directly determined by the health level of residents in the region, namely:

(2) $$\begin{array}{r} M_{t}^{i}\;=\text{ ∅}R_{t}^{i} \end{array}$$

Where $R_{t}^{i}$ represents the health level of residents in period *t*, ∅ represents the transformation coefficient of residents' health level to healthy human capital, 0 < ∅ < 1.

The decision of public health capital. It can be seen from Equation (1) that public health capital is also essential to the final product of society, and it complements corporate capital (25, 27). It is assumed that the public health capital is determined by the current local government's public health expenditure^11^, namely:

(3) $$\begin{array}{r} A_{t}^{i}\;=\;\delta G_{t}^{i} \end{array}$$

Where formula (3) ignores the lag of the public health capital, $G_{t}^{i}$ represents the public health investment of the local government, δ represents the conversion coefficient of the local government public health input to the public health capital $A_{t}^{i}$, 0 < δ < 1_◦_

Decision of residents' health level. It is assumed that the health level of the representative residents is determined by the residents' personal behavior and lifestyle (measured by the residents' health input) $\gamma \left(1-\pi \right)Y_{t}^{i}$ and the public health capital $A_{t}^{i}$ provided by the government. Since these inputs have a lagging effect on the health level of residents, the health level of residents in period *t* + 1 is actually determined by the residents' personal health investment and the public health investment of local governments in period *t*. Assuming that these inputs are additive as residents' health preferences, we get:

(4) $$\begin{array}{r} R_{t\;+\;1}^{i}\;=\;{\left[\gamma \left(1-\pi \right)\vartheta Y_{t}^{i}\;+\;\theta A_{t}^{i}\right]}^{\frac{1}{\rho }} \end{array}$$

Where Equation (4) means that the health of residents depends not only on the behavior of individual residents, but also on the behavior of local governments. It is assumed that both environmental pollution control and public health facilities are invested by local governments. Among them, ρ > 1 means that the transformation technology of residents' health input and public health capital into residents' health level conforms to the law of diminishing marginal returns, γ is the coefficient of residents' disposable income for health input, and π is the unified tax rate of the central government. 0 ≤ ϑ ≤ 1, 0 ≤ θ ≤ 1 respectively represent the coefficients of personal health investment and public health investment transformed into residents' health^12^. Derived from Equations (3) and (4):

(5) $$\begin{array}{r} G_{t}^{i}\;=\;\frac{\gamma \left(1-\pi \right)}{\delta }Y_{t}^{i}-\frac{1}{\text{θδ}}{\left(R_{t\;+\;1}^{i}\right)}^{\rho } \end{array}$$

Social constraints. Assuming that there is a representative resident in area *i*, his objective function is:

(6) $$\begin{array}{r} \overset{\infty }{\underset{t\;=\;0}{\sum }}{\tilde{\beta }}^{t}u\left(C_{t}^{Ci}\right) \end{array}$$

Where ${\tilde{\beta }}^{t}$ is the discount factor, and $C_{t}^{Ci}$ is the consumption of residents in area *i* in period *t*. The budget constraints faced by consumers are:

(7) $$\begin{array}{r} K_{t\;+\;1}^{i}\;=\;\left(1-\gamma \right)\left(1-\pi \right)Y_{t}^{i}-C_{t}^{Ci} \end{array}$$

Where Equation (7) means that the enterprise is fully depreciated, and the residents' disposable income is used for health expenditures and other consumption expenditures. Residents own businesses. For this reason, the problem of maximizing the utility of residents is transformed into maximizing formula (7) under the condition of satisfying constraint formula (6).

The specific form of setting the consumer utility function of region *i* is:

(8) $$\begin{array}{r} u\left(C_{t}^{Ci}\right)\;=\;lnC_{t}^{Ci} \end{array}$$

Where based on the existing conclusions of the Ramsey model and formula (1), the explicit solution of the optimal consumption function for region *i* is:

(9) $$\begin{array}{r} C_{t}^{Ci}\;=\;\left(1-\alpha {\tilde{\beta }}^{t}\right)\left(1-\gamma \right)\left(1-\pi \right)Y_{t}^{i}\left(K_{t}^{i},R_{t}^{i}\right) \end{array}$$

Where substituting formula (9) into formula (7), the social constraints faced by local officials are:

(10) $$\begin{array}{r} K_{t\;+\;1}^{i}\;=\;\alpha \left(1-\gamma \right)\left(1-\pi \right){\tilde{\beta }}^{t}Y_{t}^{i} \end{array}$$

Local government budget constraints. It is assumed that the local government of region *i* in period *t* has only two kinds of income: one is to levy taxes according to the unified tax rate π of the central government to obtain local fiscal revenue $\pi Y_{t}^{i}$ (ignoring fiscal share)^13^; the other is to have the initial fiscal revenue $S_{0}^{i}$. There are two types of expenditures by the local government in area *i* during the *t* period: one is the local government's own consumption expenditure $C_{t}^{Ri}$; the other is the public health investment expenditure $G_{t}^{i}$ Assuming that the local government implements a balanced budget, then the budget constraint of the local government is:

(11) $$\begin{array}{l} G_{0}^{i}\;+\;C_{t}^{Ri}\;=\;S_{0}^{i}\;+\;\pi {\bar{P}}_{0}^{i}Y_{0}^{i};\\ \;G_{t}^{i}\;+\;C_{t}^{Ri}\;=\;\pi {\bar{P}}_{t}^{i}Y_{t}^{i} \end{array}$$

Where the price $P_{t}^{i}$ is normalized to 1. Equation (11) means that the local government's public health investment (including environmental pollution control investment) $G_{t}^{i}$ is recovered through taxation, and it has initial fiscal revenue in the *t* = 0 period.

#### Benchmark Model: Consumption Preference

The utility of local officials. As the leaders of local governments, local officials directly prefer the local government's own consumption $C_{t}^{Ri}$ during the term of office, which means that local officials have economic incentives. Then the utility function of local officials is expressed as:

(12) $$\begin{array}{r} U^{i}\;=\;\overset{T}{\underset{t\;=\;1}{\sum }}\beta^{t}C_{t}^{Ri} \end{array}$$

Where *T* ≥ 2 and bounded, means that the term of office of local officials is more than one year, *t* = 1, 2, ⋯ , *T*;β is the discount factor of local officials.

The best behavior of local officials. From Equation (12) and constraint Equations (5, 10, 11), the optimization problem can be expressed as:

(13) $$\begin{array}{r} \underset{\left\{A_{t\;+\;1}^{i}\right\}}{Max}\overset{T}{\underset{t\;=\;0}{\sum }}\beta^{t}\left[\Phi Y_{t}^{i}\;+\;\frac{1}{\text{θδ}}{\left(R_{t\;+\;1}^{i}\right)}^{\rho }\right] \end{array}$$

$s.t.K_{t\;+\;1}^{i}\;=\;m{\tilde{\beta }}^{t}Y_{t}^{i}\;$Where set *m* = α(1 − π)(1 − γ), $\Phi \;=\;\pi -\frac{\gamma \left(1-\pi \right)}{\delta }$, and the end point condition is $G_{T}^{i}\;=\;0$ that is $R_{T\;+\;1}^{i}\;=\;0$. Then the Lagrangian function faced by local officials is:

(14) $$\begin{array}{r} Q^{i}\;=\;\overset{T}{\underset{t\;=\;0}{\sum }}\beta^{t}\left\{\Phi Y_{t}^{i}\;+\;\frac{1}{\text{θδ}}{\left(R_{t\;+\;1}^{i}\right)}^{\rho }\right\}\;\\ \;+\overset{T}{\underset{t\;=\;0}{\sum }}\beta^{t}\mu_{t}\left[m{\tilde{\beta }}^{t}Y_{t}^{i}-K_{t\;+\;1}^{i}\right] \end{array}$$

The optimality condition is derived from Equation (14):

(15) $$\begin{array}{r} \frac{\partial Q^{i}}{\partial R_{t\;+\;1}^{i}}\;=\;\frac{\rho }{\theta \delta }{\left(R_{t\;+\;1}^{i}\right)}^{\rho -1}\;+\;\beta \Phi \frac{\partial Y_{t\;+\;1}^{i}}{\partial R_{t\;+\;1}^{i}}\\ \;+\beta m{\tilde{\beta }}^{t\;+\;1}\mu_{t\;+\;1}\frac{\partial Y_{t\;+\;1}^{i}}{\partial R_{t\;+\;1}^{i}}\;=\;0 \end{array}$$

(16) $$\begin{array}{r} \frac{\partial Q^{i}}{\partial K_{t\;+\;1}^{i}}\;=\;-\mu_{t}\;+\;\beta \Phi \frac{\partial Y_{t\;+\;1}^{i}}{\partial K_{t\;+\;1}^{i}}\\ \;+\beta \mu_{t\;+\;1}m{\tilde{\beta }}^{t\;+\;1}\frac{\partial Y_{t\;+\;1}^{i}}{\partial K_{t\;+\;1}^{i}}\;=\;0 \end{array}$$

(17) $$\begin{array}{r} \frac{\partial Q^{i}}{\partial \mu_{t}}\;=\;m{\tilde{\beta }}^{t}Y_{t}^{i}-K_{t\;+\;1}^{i}\;=\;0 \end{array}$$

Where from Equations (15–17), Equations (1) and (2), we further deduced:

(18) $$\begin{array}{r} \frac{\rho }{\beta m{\tilde{\beta }}^{t}\theta \delta \omega }\frac{{\left(R_{t}^{i}\right)}^{\rho }}{Y_{t}^{i}}\;+\;\frac{\Phi }{m{\tilde{\beta }}^{t}}-\frac{\alpha \rho }{\theta \delta \omega }\frac{{\left(R_{t\;+\;1}^{i}\right)}^{\rho }}{K_{t\;+\;1}^{i}}\;=\;0 \end{array}$$

(19) $$\begin{array}{r} K_{t\;+\;1}^{i}\;=\;m{\tilde{\beta }}^{t}Y_{t}^{i} \end{array}$$

Where Equations (18) and (19) constitute the dynamic system of the maximum utility of local officials in this situation^14^. When the system is in a stable state, $R_{t\;+\;1}^{i}\;=\;R_{t}^{i},K_{t\;+\;1}^{i}\;=\;K_{t}^{i},Y_{t\;+\;1}^{i}\;=\;Y_{t}^{i}$, by formula (18) and (19), the equilibrium state of the system can be solved as:

(20) $$\begin{array}{r} {\left(R^{i}\right)}^{\rho }\;=\;\frac{\beta \theta \omega \left[\gamma \left(1-\pi \right)-\text{πδ}\right]}{\rho \left(1-\alpha \beta \right)}Y^{i} \end{array}$$

Where the economic meaning of Equation (20) is very intuitive. In a steady-state economy, there is a stable equilibrium relationship between residents' health and regional output.

#### Extended Model: People's Livelihood Preference of Local Officials

The utility of local officials. In a politically centralized and economically decentralized economy, local officials, as leaders of local governments, must consider their own consumption of $C_{t}^{Ri}$ during their term of office, indicating that local officials have economic incentives; and they must also pay attention to the health of residents, invest in public health infrastructure and control environmental pollution for implement the central planner's “people-centered” politics requirement. This indicates that local officials have a preference for people's livelihood. Compared with the benchmark model, according to the requirements of the central planner, local officials have both economic incentives and people's livelihood preferences. If these utilities are additive, the utility function of local officials is expressed as:

(21) $$\begin{array}{r} U^{i}\;=\;\overset{T}{\underset{t\;=\;1}{\sum }}\beta^{t}\left[\lambda C_{t}^{Ri}\;+\;\left(1-\lambda \right)G_{t}^{i}\right] \end{array}$$

Where *T* ≥ 2 and bounded, which means that the term of office of local officials is more than one year, *t* = 1, 2, ⋯ , *T*;β is the discount factor of local officials; 0 < λ ≤ 1 means the relative importance of local officials on local government's own consumption, the larger λ is, the more local officials prefer the government's own consumption. When λ = 1, it means that local officials completely prefer government consumption, regardless of the health of local residents. The smaller λ is, the more local officials prefer the health of local residents. Suppose λ > 0^15^.

The best behavior of local officials. From Equation (21) and constraint Equations (5,10,11), the optimization problem can be expressed as:

(22) $$\begin{array}{r} \underset{\left\{A_{t\;+\;1}^{i}\right\}}{Max}\overset{T}{\underset{t\;=\;0}{\sum }}\beta^{t}\left[\Phi_{1}Y_{t}^{i}-\frac{\left(1-2\lambda \right)}{\text{θδ}}{\left(R_{t\;+\;1}^{i}\right)}^{\rho }\right]\\ s.t.K_{t\;+\;1}^{i}\;=\;m{\tilde{\beta }}^{t}Y_{t}^{i} \end{array}$$

Where set *m* = α(1 − π)(1 − γ), $\Phi_{1}\;=\;\lambda \pi +\;\left(1-2\lambda \right)\frac{\gamma \left(1-\pi \right)}{\delta }$, and the end point condition is $G_{T}^{i}\;=\;0$ that is $R_{T\;+\;1}^{i}\;=\;0$. Then the Lagrangian function faced by local officials is:

(23) $$\begin{array}{r} Q^{i}\;=\;\overset{T}{\underset{t\;=\;0}{\sum }}\beta^{t}\left\{\Phi_{1}Y_{t}^{i}-\frac{\left(1-2\lambda \right)}{\text{θδ}}{\left(R_{t\;+\;1}^{i}\right)}^{\rho }\right\}\\ \;+\overset{T}{\underset{t\;=\;0}{\sum }}\beta^{t}\mu_{t}\left[m{\tilde{\beta }}^{t}Y_{t}^{i}-K_{t\;+\;1}^{i}\right] \end{array}$$

The optimality condition is derived from Equation (23):

(24) $$\begin{array}{r} \frac{\partial Q^{i}}{\partial R_{t\;+\;1}^{i}}\;=\;-\frac{\left(1-2\lambda \right)\rho }{\text{θδ}}{\left(R_{t\;+\;1}^{i}\right)}^{\rho -1}\;+\;\beta \Phi_{1}\frac{\partial Y_{t\;+\;1}^{i}}{\partial R_{t\;+\;1}^{i}}\\ \;+\beta m{\tilde{\beta }}^{t\;+\;1}\mu_{t\;+\;1}\frac{\partial Y_{t\;+\;1}^{i}}{\partial R_{t\;+\;1}^{i}}\;=\;0 \end{array}$$

(25) $$\begin{array}{r} \frac{\partial Q^{i}}{\partial K_{t\;+\;1}^{i}}\;=\;-\mu_{t}\;+\;\beta \Phi_{1}\frac{\partial Y_{t\;+\;1}^{i}}{\partial K_{t\;+\;1}^{i}}\\ \;+\beta \mu_{t\;+\;1}m{\tilde{\beta }}^{t\;+\;1}\frac{\partial Y_{t\;+\;1}^{i}}{\partial K_{t\;+\;1}^{i}}\;=\;0 \end{array}$$

(26) $$\begin{array}{r} \frac{\partial Q^{i}}{\partial \mu_{t}}\;=\;m{\tilde{\beta }}^{t}Y_{t}^{i}-K_{t\;+\;1}^{i}\;=\;0 \end{array}$$

Where from Equations (24–26) and Equation (3), we can further introduce:

(27) $$\begin{array}{r} -\frac{\left(1-2\lambda \right)\rho }{\omega \theta \delta \beta m{\tilde{\beta }}^{t}}\frac{{\left(R_{t}^{i}\right)}^{\rho }}{Y_{t}^{i}}+\frac{\Phi_{1}}{m{\tilde{\beta }}^{t}}+\frac{\alpha \left(1-2\lambda \right)\rho }{\omega \text{θδ}}\frac{{\left(R_{t\;+\;1}^{i}\right)}^{\rho }}{K_{t\;+\;1}^{i}}\;=\;0 \end{array}$$

(28) $$\begin{array}{r} K_{t\;+\;1}^{i}\;=\;m{\tilde{\beta }}^{t}Y_{t}^{i} \end{array}$$

Where Equations (27) and (28) constitute the dynamic system of the maximum utility of local officials in this situation^16^. When the system is in a stable state, $R_{t\;+\;1}^{i}\;=\;R_{t}^{i}$, $K_{t\;+\;1}^{i}\;=\;K_{t}^{i}$, $Y_{t\;+\;1}^{i}\;=\;Y_{t}^{i}$, by formula (27) and (28), the equilibrium state of the system can be solved as:

(29) $$\begin{array}{r} {\left(R_{t\;+\;1}^{i}\right)}^{\rho }\;=\;\left[\frac{\lambda \pi \delta \beta \theta \omega }{\rho \left(1-\alpha \beta \right)\left(1-2\lambda \right)}\;+\;\frac{\gamma \beta \theta \omega \left(1-\pi \right)}{\rho \left(1-\alpha \beta \right)}\right]Y^{i} \end{array}$$

Where Equation (29) reveals the intuitive economic meaning. In a steady-state economy, there is a stable equilibrium relationship between residents' health and regional output. Equation (29) means that 1 − 2λ cannot be equal to 0, otherwise Equation (29) is mathematically meaningless.

#### Discussion and Summary Analysis

In the benchmark model, formula (20) reveals: when γ − πγ − πδ > 0, the output will have a positive impact on the health of residents; when γ − πγ − πδ < 0, the output will have a negative impact on the health of residents, that is, whether the impact of output on the health of residents is positive or negative depends on the degree to which residents pay attention to health input γ, the central government's unified tax rate π, and the government's public health input conversion coefficient δ. This is also the fundamental reason and internal mechanism of the uncertainty about the impact of economic growth on the health of residents in the current academic circles.

In the extended model, formula (29) reveals that when the *Y*^*i*^ coefficient $\frac{\lambda \pi \delta \beta \theta \omega }{\rho \left(1-\alpha \beta \right)\left(1-2\lambda \right)}\;+\;\frac{\gamma \beta \theta \omega \left(1-\pi \right)}{\rho \left(1-\alpha \beta \right)}>0$, that is, when λπδ > (1 − 2λ)[1 − γ(1 − π)], the output has a positive impact on residents' health, when λπδ < (1 − 2λ)[1 − γ(1 − π)], the output has a negative impact on the health of residents. Furthermore, when 1 − 2λ > 0, that is, $\lambda <\frac{1}{2}$, the output will have a significant positive impact on residents' health. In other words, when (1 − λ)− λ > 0, that is, when the local government's relative importance to people's livelihood (1 − λ) is greater than its own consumption λ, the output will have a significant and positive impact on residents' health, which proved mathematically that the people's livelihood incentives of central planners promoted the improvement of residents' health^17^. This work means that the improvement of residents' health depends not only on the total amount of available resources brought about by economic growth, but also on the allocation of available resources by the government, which is consistent with the conclusions of the existing literature (2).

Comparison between the extended model and the benchmark model: due to the existence of local government's livelihood preferences, in a stable state, when 1 − 2λ > 0, no matter how the other parameters are within the effective limits, the impact of output on residents' health in the extended model is significantly higher than that in the baseline model, namely $\frac{\lambda \pi \delta \beta \theta \omega }{\rho \left(1-\alpha \beta \right)\left(1-2\lambda \right)}\;+\;\frac{\beta \theta \omega \gamma \left(1-\pi \right)}{\rho \left(1-\alpha \beta \right)}>\frac{\beta \theta \omega \left[\gamma \left(1-\pi \right)-\pi \delta \right]}{\rho \left(1-\alpha \beta \right)}$, revealing the importance and special significance of local government's livelihood preference for residents' health.

Extreme case: when λ = 1, the extended model situation is transformed into the baseline model situation, and the equilibrium relational Equation (29) is transformed into Equation (20). When λ becomes smaller and smaller from 1, that is, when local officials have more and more preference for the health of residents in the region, the coefficient of *Y*^*i*^ in Equation (29) will become larger and larger, which means that the output is more effective for residents' health. The impact is getting bigger and bigger.

Furthermore, the previous analysis provides a theoretical basis for follow-up empirical research. Taking into account the “people-oriented” and later “people-centered” political and people's livelihood requirements put forward by China's central planners since 2002, we believe that local officials pay more attention to people's livelihood preferences than to local governments' own consumption. For this reason, the hypothesis that economic growth promotes the health of Chinese residents based on people's livelihood preference is proposed. Taking the Logarithm on both sides of Equation (29), we get:

(30) $$\begin{array}{r} lnR_{t\;+\;1}^{i}\;=\;\varpi \;+\;\frac{1}{\rho }lnY^{i} \end{array}$$

Where $\varpi \;=\;\frac{1}{\rho }\ln\frac{\lambda \pi \delta \beta \theta \omega +\;\gamma \beta \theta \omega \left(1-\pi \right)\left(1-2\lambda \right)}{\rho \left(1-\alpha \beta \right)\left(1-2\lambda \right)}$, since ρ > 1, the region output is positively correlated with the health level of residents, revealing the importance of local officials' livelihood incentives for residents' health.

## Empirical Analysis

### Methodology

Based on Equation (30), this study expresses the econometric model of the hypothesis that economic growth promotes the health of Chinese residents as:

(31) $$\begin{array}{r} lnR_{it}\;=\;a_{0}\;+\;a_{1}lnY_{it}\;+\;a_{2}lnX_{it}\;+\;u_{it} \end{array}$$

Where *a*_0_ is a constant term, *u*_*it*_ is a disturbance term, which obeys the standard logarithmic normal distribution. *R*_*it*_ represents the health level of residents in each province (city). *Y*_*it*_ is regional output. *X*_*it*_ represents a series of control variables.

It is worth noting that there are three points in formula (31) that are worth emphasizing: First, when the hypothesis of “economic growth promotes the health of Chinese residents” is established, *a*_1_ > 0, for this reason, it is most concerned about the regression coefficient in this study. The second is that Equation (31) may have endogenous problems, that is, residents' health and regional output may be endogenous. Third, because the theoretical analysis did not consider the influence of population factors, the relevant variables should be averaged or averaged (except for the ratio-based control variables) in the empirical study.

### Data

We selects the panel data of 31 inland provinces (cities) in China from 2004 to 2019 for analysis. The main reason is the limitation of data. The selection of provinces as the analysis unit is based on the fact that provincial governments have played a major role in public health investment in recent years, and local governments' public health investment accounts for more than 70% of the total expenditure of governments' public health investment (1). The regression estimation sample started from 2004, mainly considering that the SARS occurred in 2002–2003 was an outlier year and Comrade Hu Jintao put forward the “people-oriented” scientific development concept in July 2003, and its policy effects should occur in 2004. The sample ended in 2019 mainly because the latest data to measure the health level of residents is only up to 2019. No newer data has been found and the data of some control variables faces the same problem. In addition, it is also considered that the impact of COVID-19 in 2020 is abnormal. The specific data selection and processing are as follows^18^.

The explained variable resident health level *R* is measured by proportion of moderate to severe malnutrition in children under 5 years old (%) in 31 inland provinces^19^. Zhong et al. (29) adopted a similar measurement method. Because the indicator is negative index to resident health, we take the reciprocal of the index, so that the negative impact will transform the positive impact, and Yang et al. (30) adopts a similar processing method. Then we perform logarithmic processing, which is recorded as *lnR*.

The explanatory variable output Y is measured by the per capita GDP of each province. In order to eliminate the general price trend, we use the per capita GDP index of each province at constant prices (2004 = 100) to eliminate the impact of price changes, and take the logarithm and record it as *lnGDPper*.

The selection of other control variables follows the idea of Zhao and Jin (31) combing the mechanism of economic growth on public health. Economic growth can not only directly affect public health, but also through education, healthcare, urbanization, industrialization, etc. indirectly have an impact on public health. In addition, economic growth may also regulate the effects of education levels affecting public health. To this end, the control variables are selected as follows: Urbanization, which is measured by dividing the urban population by the total population, which is recorded as *URR*. Industrialization, which is measured by the industrialization rate, which is the proportion of industrial added value in the current year's GDP, and it is recorded as *INR*. The level of education development, which is measured by the ratio of teachers to students in ordinary colleges and universities (the number of teachers = 1) and recorded as *EDL*; the unemployment situation is measured by the unemployment rate of the urban population and recorded as *UR*. Descriptive statistics are shown in Table 1.

**Table 1:** Descriptive statistics of related variables.

**Variable**	**Mean**	**Std. Dev**.	**Min**	**Max**	**Obs**
Resident health logarithmic value (*lnR*)	4.434	0.9177863	2.629701	7.418581	496
Per capita GDP log value (*lnGDPper*)	10.14724	0.6794547	8.353262	11.97832	496
Urbanization rate (URR)	0.5252978	0.1478545	0.2071429	0.9415162	496
Industrialization rate (INR)	35.36537	9.818246	7.04698	57.3781	496
Educational Development Level (EDL)	17.21829	1.261598	10.79	21.34	496
Unemployment rate (UR)	3.453024	0.7426617	0	6.5	496

### Preliminary Estimation of the Model

In order to obtain reliable results, the fixed-effects F test and the random-effects Hausman test were first performed. The results showed that the null hypothesis was rejected. For this reason, fixed effects regression (FE) was selected for estimation. The regression results of the model are shown in Table 2 Equation (1). From this equation, it can be seen that the hypothesis that economic growth promotes the health of Chinese residents is significant, and *a*_1_ > 0. In addition, it can be seen from the control variables that the urbanization rate, industrialization rate, and education development level are positively correlated with residents' health, while the unemployment rate is negatively correlated with residents' health. These situations are in line with reality.

**Table 2:** Regression results.

**Explanatory variables**	**Panel fixed effects model**		**Consider endogenous issues(FE)**	
	**Equation (1)** **FE**	**Equation (2)** **Driscoll-Kraay**	**Equation (3)** **IVFE**	**Equation (4)** **FEGMM**
*lnGDPper*	0.29767*** (0.06445)	0.29767*** (0.07486)	0.20859*** (0.07547)	0.21157*** (0.07546)
URR	1.66082*** (0.46571)	1.66082*** (0.56972)	2.33103*** (0.57312)	2.31246*** (0.57309)
INR	0.02100*** (0.00333)	0.02100*** (0.00264)	0.02174*** (0.00357)	0.02176*** (0.00357)
EDL	0.02211 (0.01467)	0.02211 (0.01695)	0.00273 (0.01657)	0.00275 (0.01657)
UR	−0.0737** (0.03019)	−0.0737** (0.02191)	−0.0881*** (0.03166)	−0.0883*** (0.03166)
cons	−0.3281 (0.56608)	−0.3281 (0.61897)	–	–
Excluded instruments	–	–	L.lnGDPper, L.URR, L.INR, L.EDL	Same as Equation (3)
Under identification test	–	–	430.96 (*p* = 0.00)	430.998 (*p* = 0.00)
Weak identification test	–	–	1.5e + 04	1.5e + 04
Sargan statistic	–	–	E3.442 (*p* = 0.3283)	5.389 (*p* = 0.1454)
Endogeneity test	–	–	10.014 (*p* = 0.0016)	10.014 (*p* = 0.0016)
R-sq	0.5463	0.4594	0.3892	0.3893
Obs	496	496	496	496

The numbers in parentheses are standard deviations. The Equation (2) is the robust standard deviation of Driscoll-Kraay. Equations (3) and (4) are estimated under the command of Sata software xtivreg2, and the numbers in parentheses (except the test items) are the standard deviations of heteroscedasticity and autocorrelation robustness (HAC). The constant term is not reported. ***, **, * are two-tailed tests that are significant at the significance levels within 1%, 5%, and 10%, respectively.

However, after autocorrelation and heteroscedasticity tests, we found that Equation (1) has first-order autocorrelation and heteroscedasticity, so the result estimated with beta standard deviation is actually biased. For this reason, the robust Driscoll-Kraay standard deviation is used to make a revised estimate, and Equation (2) is obtained. From the revised equation, it can be seen that the hypothesis that economic growth promotes the health of Chinese residents is still significant, and *a*_1_ > 0. This means that after solving the problems of autocorrelation and heteroscedasticity, the hypothesis that economic growth promotes the health of Chinese residents still has significant explanatory power.

### Solving Endogenous Problems

According to the previous analysis, in the regression Equation (31), the residents' health level and output may be endogenous. For this reason, the explanatory variable *lnGDPper* may be endogenous under certain conditions. In fact, the preliminary estimation Equations (1) and (2) in Table 2 ignore the possible endogenous problems of Equation (31), which means that Equations (1) and (2) may have biased and inconsistent regression results. In order to solve the endogenous problem, the following further tests and estimates are made.

First, the variables of Equation (2) in Table 2 are tested for endogeneity. In the estimation performed under the command of Stata software xtivreg2, the instrumental variables selected for the possible endogenous variable *lnGDPper* include its one-period lag value and the one-period lag value of *URR*, *INR* and *EDL*. The endogenous test of *lnGDPper* shows rejection of the null hypothesis, indicating that *lnGDPper* is an endogenous variable, which is consistent with the expectations of the previous theoretical analysis, and the selected instrumental variables passed the validity test. Exogenous tests were performed on *URR*, *INR*, *EDL*, and *UR* variables, and the results showed that the null hypothesis was accepted^20^.

Second, in order to overcome the endogenous problem, the instrumental variable fixed effect estimation (IVFE) is carried out according to the selection of endogenous variables above. The specific situation is shown in Table 2 Equation (3). The results show that the estimation is effective and reliable, and *a*_1_> 0.

Third, the panel fixed-effect GMM estimation is used to further confirm the robustness of the estimation results. Similarly, the HAC robust standard deviation is used to estimate the GMM of Equation (2). The specific situation is shown in Table 2 Equation (4). From the results of GMM estimation, it is very close to the result of IVFE estimation, which also shows the existence of model robustness. In addition, except for *UR*, all other explanatory variables are significant, and the positive and negative directions of the variable coefficients are in line with economic reality, which shows that the choice of model control variables basically meets the initial expectations, and also verifies the robustness of the model from the side.

Finally, examine the impact of different instrumental variables on the results, all instrumental variables pass the validity test (specific analysis is not reported).

In summary, the estimation results in Table 2 are valid and reliable, and the hypothesis that economic growth promotes the health of Chinese residents is established.

## Conclusion

The impact of economic growth on residents' health depends not only on residents' personal health investment and protection, but also on government public health investment behavior. This paper proves mathematically that in an economy with economic decentralization and political centralization, as local governments implement the central planner's requirements of “people-oriented” and “people-oriented,” local officials must pay attention to not only their own consumption but also residents' health. Therefore, it is necessary to increase investment in public health facilities and environmental pollution control, resulting in the demand for people's livelihood, and the conclusion is that there is a stable and balanced relationship between economic growth and residents' health. When the local government's relative importance to people's livelihood is greater than its own consumption, the economic growth will have a significant and positive impact on residents' health. And the internal mechanism of economic growth affecting residents' health is combed mathematically. Using panel data from 31 inland provinces from 2003 to 2019, empirical tests show that the hypothesis that China's economic growth promotes residents' health is established. The work of this article means that the improvement of residents' health depends not only on the total amount of available resources brought about by economic growth, but also on the allocation of available resources by the government. The Chinese local government's investment in public health facilities and environmental pollution control is an important clue to explain which China's economic growth can promote the improvement of residents' health. The policy implication is that whether it is the central planner or the local government, increasing investment in public health is an important and key policy direction for improving the health of residents, and they should strengthen the investment of available resources in public health infrastructure and increase efforts to control environmental pollution in order to achieve the goal of improving the health of local residents.

## Data Availability Statement

The original contributions presented in the study are included in the article/Supplementary Material, further inquiries can be directed to the corresponding author/s.

## Author Contributions

S-tC: writing-original draft, conceptualization and methodology, software, and data preparation. W-hY: writing and reviewing. All authors contributed to the article and approved the submitted version.

## Funding

This research was partly supported by General Project of 2021 Shanghai Party Institute of CPC (School of Administration) System (2021SHB027) and Philosophy and Social Sciences Fund of Guangxi Province (21BYJ021).

## Conflict of Interest

The authors declare that the research was conducted in the absence of any commercial or financial relationships that could be construed as a potential conflict of interest.

## Publisher's Note

All claims expressed in this article are solely those of the authors and do not necessarily represent those of their affiliated organizations, or those of the publisher, the editors and the reviewers. Any product that may be evaluated in this article, or claim that may be made by its manufacturer, is not guaranteed or endorsed by the publisher.

## Footnotes

^1^The data comes from the China Statistical Yearbook and official data from the China Health Commission.

^2^Since the reform and opening up, the average annual growth rate of government health investment has been higher than the average annual growth rate of fiscal expenditure, but the proportion of government health investment in total health expenditure has been declining year by year. Government investment in public health facilities has gone from full budget management before the reform and opening up, to a decline in the proportion of budgetary allocations and a gradual increase in extra-budgetary funds after the reform and opening up, until the increase in investment after 2003; while the government's basic medical investment has gone through In the planned economy period, “supply and demand are both supplemented and supplemented mainly” to “demand-side, purchasing services” under the market economy system, and tilted towards urban and rural primary health institutions (1).

^3^The public health investment data from 2018 to 2020 are estimates, based on the year-on-year growth rate of fixed asset investment in the health sector that year. In addition, there is a lack of investment in environmental pollution control in 2018-2020. Public health input data is the number of fixed asset investments (excluding rural households) in the health, social security and social welfare industries.

^4^Even if it is empirical research, domestic research is mostly limited to simple, general empirical research that lacks the support of stable theoretical relationships. Most of them adopt time series numbers, which are prone to missing variables, leading to deviations in research results.

^5^The public health investment in this study specifically refers to the local government's investment in environmental pollution control and public health facilities investment, both of which are considered together.

^6^What needs to be pointed out here is that even if the central government implements investment in environmental pollution control and public health facilities, it is implemented through local governments. They transfer funds to local governments, and then require local governments to organize implementation, which is essentially a financial share problem.

^7^What needs to be pointed out here is that in the existing Western economics literature, the government's objective function is usually set to maximize social welfare (that is, to maximize the sum of producer surplus and consumer surplus), because the government is democratically elected Produced, the government represents public opinion. But the environment set up in this article is political centralization and economic decentralization. In a politically centralized environment, on the one hand, the local government's decision-making is usually the “top leader” in charge, and the local government is actually “kidnapped” by local officials. For this reason, the actions of local governments do not necessarily represent public opinion, but rather the preferences of local officials. In other words, local governments are actually represented by local officials, and the decision-making behavior of local governments is “personified” by local officials. Therefore, the local government's own consumption expenditure has the color of local officials. On the other hand, in a politically centralized environment, an important aspect of the promotion and evaluation of local officials is determined by votes through internal government staff (usually referred to as “democratic evaluation”). For this reason, if local officials want to obtain promotion opportunities, they must pay attention to the self-interests of the internal staff of the local government, and the local government's own consumption expenditure is directly linked to the interests of the internal staff of the local government. Therefore, whether it is from the perspective of local government consumption that has the color of local officials, or from the perspective of local officials' promotion and assessment incentives, the objective function of local officials prefers the local government's own consumption.

^8^Economic incentives are expressed as local officials' pursuit of maximizing local government's own consumption. It is the most basic incentive mechanism for local officials. People's livelihood incentives are expressed as local officials' public health investment preferences, which are usually highly related to the requirements of the central government.

^9^Other human capital investment such as education and high school by doing is ignored here.

^10^α + ω + φ < 1 Means that the production of representative firms is assumed to be diminishing returns to scale.

^11^The lag effect of the construction of public facilities is ignored here.

^12^Equations (1, 4) means that government investment in public health facilities can either directly affect output through public health facility capital, or indirectly affects output by affecting residents' health and thus healthy human capital.

^13^What needs to be pointed out here is that the central government's financial transfer support to local governments is also an important part of the local government's public health input. However, because the central government's financial transfer payments also come from taxes, they are actually a part of the output $Y_{t}^{i}$ fixed ratio. For this reason, this study ignores the factor of central fiscal payment.

^14^According to the existing conclusions of Cen (26), it can be concluded that the dynamic system has a saddle-shaped stable path, so the system is stable.

^15^If λ = 0, so it is possible that local government consumption is negative, so it is excluded.

^16^Similarly, according to the existing conclusions of Cen (26), it can be concluded that the dynamic system has a saddle-shaped stable path, so the system is stable. Ge and Cen (28) have concluded that in the same way, a stable state can be promoted.

^17^Equation (29) reveals the uncertainty of the impact of economic growth on residents' health. If people's livelihood incentives are ignored, then economic growth may have a negative impact on residents' health. This is one of the reasons for the constant debate on the relationship between economic growth and residents' health in the current literature. It refutes the view that “as long as economic growth goes up, other aspects including health will go up naturally”, and proves that “the improvement of residents' health depends on both available resources amount and distribution of resources”.

^18^Except for specially marked sources, the data all come from the national statistical database of China.

^19^The original data of this variable comes from the China Economic and Social Big Data Research Platform of CNKI. The data for 2006 and 2009 are missing, and they were replaced by the mean of the data for the previous and subsequent years.

^20^For the sake of robustness, we also used the Davidsom-MacKinnon endogeneity test method under fixed-effect conditions to test the variable, and the results are consistent.
