# Supplementary material for: Economic Growth, People's Livelihood Preferences of Local Governments and Residents' Health
Source: Front Public Health. 2022 Apr 8;10:844015. doi: 10.3389/fpubh.2022.844015 (PMC9024032; doi:10.3389/fpubh.2022.844015)
Supplement: Supplementary file 1 [file Data_Sheet_1.docx]

**Attached table of test:**

**1. F** **test**

**2. Housman test**

**3****.** **Fixed effects model regression results**

**4.** **Autocorrelation test**

**5.** **Heteroscedasticity test**

**6.** **FE regression results with robust Driscoll-Kraay standard deviation**

**7.** **Endogenous test**

**8.** **Exogenous test**

**（1）****test URR**

**（2）test INR**

**（3）test EDL**

**（4）test UR**

**8. IVFE** **estimation**

**9. FEGMM** **estimation**
